# A Dictionary of Practical Medicine

**Published:** 1835-07-01

**Authors:** 


*A Dictionary of Practical Medicine.* By JAMES COPLAND, M.D. F.R.S. &c. Part III.—London, 1835. 8vo. pp. 320.

DR. COPLAND'S admirable work, of which the third part is now before us, is so condensed in style, and so excellent in execution, that analysis is impossible, and criticism very difficult. In such a case as this, therefore, we decline the functions of censor, content with the more agreeable office of indicator. The present part is fully equal to the preceding ones; and we agree with the whole world in thinking Dr. Copland's Dictionary not only superior to any existing one, but likely long to remain at the head of its class:

————maturos largimur honores,
Nil oriturum aliàs, nil ortum tale fatentes.

This part contains forty-nine articles, from Dropsy of the Pericardium to Remittent Fever, including Dysentery, Epilepsy, Erysipelas, and Diseases of the Eye. We give a few extracts, rather in compliance with custom, than with the hope of giving our readers a notion of the work: the brick in the story could not have been a more unsatisfactory sample of the house. This is of the less importance, however, as, sooner or later, every one will get the book; and this is an additional reason for making but scanty quotations.

*Practice of the German Physicians in Acute Hydrocephalus.*

"The following is an abstract, made in my note book many years since, of the practice of the most able German physicians in this disease. In the nervous or typhoid variety, cold applications to the head, sinapisms to the arms and legs, and purgative clysters. If these fail, digitalis, with the decoction of flores arnicæ or infusion of serpentaria; blisters from the occiput to between the shoulders to be kept open, the inunction of mercury three or four times a day; and, if the vomiting persist, sinapisms on the epigastrium. Subsequently musk and ammonia, chiefly on account of the convulsions. In the inflammatory form, and in that consequent on the exanthemata, local bleedings, digitalis, calomel and jalap, and in the latter stages of these forms, the treatment directed for the nervous or typhoid variety." (P. 675, *note*.)

We can afford room for the conclusion only of our author's history of the following interesting operation.

*Tapping in Chronic Hydrocephalus.*

"309. β. Mr. Greatwood (*Lancet*, No. 299, p. 238,) records a case of a hydrocephalic child, æt. fifteen months, who, falling on a nail, punctured the head at the upper third of the lambdoidal suture. The wound continued to discharge fluid for several days, and it afterwards perfectly recovered from the disease. In the same work, for April and November, 1830, the operation of puncture is stated to have been successfully performed in St. Bartholomew's Hospital. Graefe (his *Journ.* for 1831, b. xv. p. 3,) punctured the head of an infant hydrocephalic from birth, in the fourth month, and repeated the operation about eleven times during six months. The fluid was allowed to escape slowly each time; the canula being removed, and the wound closed, as soon as the pulse became weak. After the last puncture, the sutures closed. The child could walk and speak when a year old. At the age of two years and a half, it was shown to the Medico-Chirurgical Society of Berlin. Mr. Russel (*Edin. Med. and Surg. Journ.* July 1832, p. 43,) operated on a girl eight months old, hydrocephalic from birth, and whose head was twenty-three inches in circumference when he first punctured it. The operation was repeated four times, after intervals of about ten days; but the quantity of fluid withdrawn each time was small. After the last puncture, calomel was given so as to affect the mouth; when the hydrocephalic symptoms disappeared, and ossification of the sutures proceeded. The case is stated to have been cured. Dr. Conquest is reported, in a contemporary work, to have operated in nine cases, successfully in four of them. The greatest number of punctures in one case were five, and the intervals between them from two to six weeks. The largest total quantity of water removed was fifty-seven ounces, by five operations; and the largest quantity at one time, twenty ounces. The trocar was introduced through the coronal suture, below the anterior fontanelle, and the wound carefully closed after each evacuation. Pressure was made by means of strips of adhesive plaster." (P. 682.)

*Pathological Effects of Calomel.*

"A most important fact was determined by the experiments performed by Mr. ANNESLEY (*Sketches of Dis. of India*, 2d ed., 8vo. p. 374), in order to ascertain the operation of calomel: and these experiments presented uniform results, viz. that, whilst the stomach and duodenum of dogs that had taken large doses of this preparation were much paler and less vascular than in ordinary circumstances, the colon and rectum, from the cæcum to the verge of the anus, were most acutely inflamed: thereby explaining the results of clinical observation; namely, that, although large doses of calomel calm those symptoms usually caused by increased vascular action, or inflammation of the mucous surface of the stomach and duodenum, they lower the vital energy of these important organs, and occasion tenesmus, griping pains in the course of the colon, mucous or bloody stools, hæmorrhoids; and, if persisted in, many more of the symptoms of dysentery, or even structural change of the colon and rectum. I am confident that dysentery becomes chronic; that an occasional indigestion lapses into a constant dyspepsia; and the habitual constipation often passes into strictures of the rectum, and hæmorrhoids into fistulæ; from the frequent exhibition of large doses of this medicine. Ingenuity cannot possibly devise a more successful method of converting a healthy person into a confirmed invalid, of destroying many of the comforts of existence, and of occasioning hypochondriasis and melancholy, than the practice of prescribing large doses of calomel on every trifling occasion, or when the bowels require gentle assistance; or because the patient erroneously supposes himself to be *bilious*, or is told so by those who should know better. The unfortunate word '*bilious*' is the scapegoat of the ignorant." (P. 731, *note*, Art. *Dysentery*.)

*Bleeding to be avoided during the Coma of Epilepsy.*

"A gentleman, residing near Portman Square, had been under my care, in the spring of 1833, for articular rheumatism. He soon recovered, and went out of town. Towards the close of the year, whilst in Scotland, he had an epileptic attack; and was blooded in the arm, and cupped soon afterwards. This was the second seizure, the first having occurred two or three years before. He returned to town immediately after this second attack; and, when I saw him, there appeared no occasion for further vascular depletion: a course of alteratives and stomachic purgatives was therefore directed. Three or four days afterwards, he had a third seizure, and was brought home in the soporose stage of the fit. I did not see him until about two hours afterwards; and then a physician, who had been called in whilst I was sent for, had him cupped largely! But, soon after the depletion, and as sensibility was returning, the paroxysm recurred. The obvious course in this case was, to have caused the patient to be removed to bed, and to have stated that nothing further was requisite in that stage of the fit until the patient had partly slept off the exhaustion; when the physician in attendance would pursue that course which his knowledge of the antecedent disorders and state of the patient would warrant.

"Whilst this was passing through the press, a man of middle size, apparently about forty, consulted me; and stated that he had been seized with the first paroxysm of the disease immediately post coïtum quinquies repetitum duabus cum puellis inter hòras perpaucas; that he had been blooded to about a pint soon afterwards, and experienced a still more severe fit about a month after the first; that the third seizure occurred about a fortnight after the second, during which he fell down and cut his head, the cut part having bled a pint at least; that his usual medical attendant, upon arriving soon after the termination of this fit, bled him largely from the arm; but that, as soon as the vein was closed, the fit recurred; and that, during the struggles, the vein broke out, and the blood was allowed to flow until two or three pints were taken in addition to the quantities lost just before. The person who accompanied him to my house, on account of his weak state, and who witnessed the paroxysms, stated that this last was most severe; and that the fit which recurred during the depletion, and which was attempted to be put a stop to by continuing the abstraction of blood until a very unusual quantity was lost (about five pints in all), was remarkably prolonged and violent. The patient is now pale and weak, with a waxy appearance of the surface; completely exhausted, physically and mentally; and constantly dreading a recurrence of the paroxysms. This case furnishes a very remarkable instance, not only of the failure of large bloodletting in arresting or shortening the fits, but also of its influence in rendering them more frequent and violent, when injudiciously prescribed." (P. 799, *note ;* Art. *Epilepsy*.)

